# Changes in medicine course curricula in Brazil encouraged by the Program for the Promotion of Medical School Curricula (PROMED)

**DOI:** 10.1186/1472-6920-8-54

**Published:** 2008-11-27

**Authors:** Patrícia Alves de Souza, Angélica Maria Bicudo Zeferino, Marco Da Aurélio Ros

**Affiliations:** 1 Graduate Program in Child and Adolescent Health of the Faculty of Medical Sciences (FCM), the State University of Campinas (UNICAMP), Campinas, Brazil; 2 Departament of Public Health, University of Santa Catarina (UFSC), Florianópolis, Brazil

## Abstract

**Background:**

The Program for the Promotion of Changes in Medical School Curricula (PROMED) was developed by the Brazilian Ministries of Health and Education. The objective of this program was to finance the implementation of changes to the curricula of medical schools directed towards the Brazilian national healthcare system (SUS). This paper reports research carried out together with the coordinators responsible for the PROMED of each medical school approved, in which interviews were used to evaluate whether this financial support succeeded in stimulating changes. The aim of this study was to evaluate the impact of this program three years after implementation in the universities that received funding.

**Methods:**

The 19 course coordinators of the medical schools in which the PROMED project was implemented were interviewed using a questionnaire containing 12 questions for qualitative analysis. This paper focuses partially on the reports of the results of this qualitative analysis. Laurence Bardin's.

**Results:**

The universities interviewed were found to have some common concerns: the decoupling of basic and professional training difficulties in achieving proximity to the network of services; insufficient funding; and the emphasis of most teachers being on teaching hospitals and specialization. These findings indicate that the direction of curriculum reform (PROMED) is toward providing a targeted training for this system.

**Conclusion:**

The interviewees were aware that this program would trigger future changes in all aspects of healthcare and represents an ongoing challenge to the academic field. PROMED provided the momentum for change in the nature of medical training in Brazil and was seen as powerful enough to override other processes and as a basis for guidance regarding the methodology, pedagogical approach and scenarios of practical experience.

## Background

In 2002, the Program for the Promotion of Changes in Medical School Curricula (PROMED) was elaborated by the Ministry of Health (MS) in partnership with the Ministry of Education (MEC) with the objective of financing curricular reform in medical schools directed towards the Brazilian national healthcare system (SUS).

The PROMED program began in 2003, when nineteen medical schools were approved for participation, after a selection process that involved more than 100 universities. The three proposed areas of reform were: theory, practice and teaching [1]. At the time of implementation no plans for an evaluation procedure had been established.

The history of curricular reform in Brazil, the context of curricular reform within the Brazilian national health system (from the Single Health System to the Family Health Program) and the conception of the PROMED program are described herein.

Brazil is a large country with about 180 million inhabitants, from which at least 80% are national healthcare system (SUS) users [2], and more than 132 medical schools[3]. Nevertheless, the profile of the graduated physician is directed toward working in private clinics whereas the process of medical training should also be designed for the public system [1].

Within a broad social movement specific to the healthcare field a new constitution was approved in 1988 (following the end of Brazil's military dictatorship), and the Brazilian national healthcare system (SUS) was created. The previous model of this healthcare system benefited the medical-industrial complex and medical training was designed to adapt to this system. Doctors were trained to work in hospitals, and in private, specialized clinics, and basic healthcare was undervalued [4].

In the early 1990s, both MEC [5] and CINAEM (the Brazilian Inter-Institutional Committee for the Evaluation of Medical Teaching) discussed this situation and found that doctors lacked the necessary competence to fulfill the demands and expectations of society [5,6].

As a result of the World Conference of Medical Education in Edinburgh, 1988, the process of medical education began to change simultaneously, in several countries, and initiatives to stimulate and support the medical teaching reform were intensified, using "*Health for Everyone in 2000" *as their health organization slogan. It was then proposed that medical schools should prepare health professionals to develop the role of helping the real situation of the Brazilian population in a system of healthcare in transformation [5]. It was only in 2001, when the curricular guidelines were finally approved that a change, in fact, was implemented in undergraduate teaching policies.

The World Health Organization recommends that there be one doctor for every 1000 inhabitants (1:1000). Although the Brazilian average is 1:594, there are some considerable regional differences between the North and Southeast regions, with ratios of 1:1190 and 1:432, respectively [7].

The total number of medical schools increased from 81 in 1995 to 125 in 2003, and the number of places offered to students had an increase of 20%, going from 7.622 to 12.081 in the same period [7].

All these changes led to the creation of PROMED, in 2002, which had the purpose of providing financial support for submitted projects, promoting and sustaining initiatives of curricular reform in medical schools that were willing to adapt their teaching methods, knowledge production and services to health requirements of the population [1,6] The focus of the program was to qualify the professionals to practice medicine in a competent, ethical and socially responsible way [8].

The projects supported by PROMED deal principally with reform in the areas of theoretical guidance, pedagogical approach and locations for practical training [1]. This reform of the medicine course should be directed by the Curricular Regulations of the Medicine Course, so that the basis of the teaching should be to provide: 1. an ethical perspective, a humanistic view, and a sense of social responsibility and commitment to the citizen; 2. orientation toward health protection and promotion and disease prevention; 3. the ability to comprehend basic knowledge and integrate and apply it in professional practice; 4. orientation toward acting at primary and secondary levels of healthcare and resolving with quality problems related to health; 5. the ability to deal with primary urgency and emergency situations; 6. the ability to communicate and deal with multiple aspects of the doctor-patient relationship; 7. the ability to continuously learn during their whole professional life and to assess their own performance; and 8. the ability to act within and later lead a health team [7]. This creates conditions for the critical and creative training of professionals able to assimilate the ideal of 'learning to learn', generating mechanisms which contribute to the permanent reconstruction of their professional identity [1].

To reorganize and stimulating basic healthcare as a strategy to substitute the traditional model, which focused on the disease and hospital-based care, requires socially-oriented and technically qualified professionals who could guarantee an effective health care to the community [8]. In the United States, the objective is to train a desirable number of physicians prepared to provide primary, basic healthcare [9].

PROMED provides resources for the implementation of the curricular guidelines approved by the Brazilian Ministry of Education (MEC) through financial support, guidance with respect to change, and flexibility in teaching procedures; in addition, it allows transformation to take place in other schools [1].

Three years after the implementation of PROMED, few evaluations of its processes had been carried out, and there was a need to assess the advances made and the difficulties encountered. The question posed was: what is PROMED's potential for promoting the desired curricular reforms? And the objective of this study was, therefore, to evaluate the impact of this program three years after its implementation in the universities that received the funding.

## Methods

Coordinators from the 19 selected universities were interviewed, and their responses were qualitatively analyzed for evidence of themes. Content analysis technique [10] was employed to evaluate the variables indicating the difficulties and potential of this project (PROMED).

The 19 course coordinators are professors who are acting as course managers, that is, they receive a salary for occupying the position of coordinator of a medicine course. The course coordinators of the medical schools in which the PROMED project was implemented were interviewed using a questionnaire containing 12 questions for qualitative analysis. This paper focuses partially on the reports of the results of this qualitative analysis. Laurence Bardin's [11] content analysis was the technique used and the methodology was based on the qualitative research of Cecilia Minayo [12].

In the first reading of the interviews, 1182 words were highlighted, as determined by the rules relating to repetition of reading, until it was possible to record them and convert them into 9 categories: evaluation, curriculum guidelines and practice, faculty, permanent education – post-graduation – research, history of the school, internship, PROMED, and health in Brazil.

The category selected for this study was curriculum guidelines and practice, and two of the proposed sub-categories were analyzed these being: a) limitations (encompassing the difficulties and problems encountered in carrying out curriculum reform); and b) challenges (description of the challenges to be faced, how they will be dealt with and what can be achieved within the perspective of reform).

To preserve anonymity, each school coordinator was identified with a reference code beginning with the letter E (educator) followed by a specific number. Interviews were transcribed and the themes described below emerged from the data. The participants read the "Free and Informed Consent Terms" and completed and signed it personally.

The project was approved by the Committee of Ethics in Research, Faculty of Medical Sciences, Campinas State University (UNICAMP), on September 27^th ^2005, project 483/2005, CAAE 1448.1.146.000-05.

## Results and discussion

### Types of universities in Brazil

In Brazil there are universities maintained by the state and federal governments (public institutions) in which free education is offered, as well are several medicine courses for which students have to pay fees (private institutions). There are also some universities partially supported by the government in which the student pays the remainder of the fee (semi-public institutions).

Another difference between the institutions is their history, some are 150 years old while others are less than 10. As a minimum curriculum is not required (according to the 1988 Constitution) the forms of teaching have also assumed a diversification, not only in terms of funding but also in terms of culture and curricular organization. Nevertheless, unexpected similarities were found in the analysis of the interviews. These common characteristics results from a hegemonic model installed in Brazil since the early sixties (Flexnerian model). Therefore, it was possible to note common concerns among the universities where the interviews were carried out: the dissociation between basic and specialist teaching; difficulties in achieving proximity with the network of services; insufficient funding and the emphasis given by the majority of professors to hospital-based specialized teaching.

These finding were observed in some interviews, for example:

**E3: **challenge – we have to consolidate this teaching in the community (...) and it is not yet adequate.

**E8: **I believe that it is not just in our school, (...) the need to reeducate the educators (...) trained according to the old model and who tend to just reproduce [material] or show resistance to implementing a new model.

**E19: **It is not sufficient that you talk to the professors and tell them that they need to refer to the healthcare network system (...) teach there (...) you have to show them that this area is absolutely indispensable for teaching medical students and other students in the field of healthcare.

These responses reveal the difficulties involved in convincing a professor to set aside the convenience of the institutional protection, which exists within the teaching hospital environment, to face the unknown. On the other hand, the current emphasis on the organization of healthcare in Brazil focuses on the Family Health Program, which is the foundation of the basic healthcare provided by the public health system (SUS). This program defends an ideal composed of the propositions of SUS such as: integrality, universality, equity, social control and hierarchical formation of the system. The principal objective of the curricular reform supported by PROMED is to promote training directed towards satisfying the requirements of this system.

These propositions have an undisclosed multiplicity of connotations that result in different meanings being attributed to the same term and consequently also in diversity in the forms of implementing changes. Terms such as equity, social control or integrality may be interpreted in different ways:

**E2: **the doctor who is integrated in the current situation with respect to citizenship...in an integrated way and who clearly understands the objectives that the curriculum reform asks of us.

**E4: **one of the fundamental conditions for this curriculum to work is its interdisciplinary nature...the principal focus being on the healthcare and health requirements of our population.

**E10: **based on [health] prevention and promotion.

Therefore, three different comprehensions affect the directionality of the curricular change. The interviews highlighted two categories from the analysis: limitations and challenges.

### Limitations

This category includes obstacles that hinder implementation of the curriculum, ranging from faculty integration to curricular organization. There is a set of various grouped components that will be discussed below:

The integration of people (members of the faculty with those of the healthcare network and the community) and contents is a critical aspect of the curriculum.

**E1: **I think there still remains a lot to be integrated in this integrated curriculum

**E7: **After several years of discussions and seminars, forums, we have finally reached a diagnosis of the course, which was that there was no basic-clinical interaction.

**E18: **We are having a lot of difficulty with respect to integration with the community.

**E17: **When we started (...), there was a group that resisted a little. These proposals involve a lot of work in that they require meetings (...) with a lot of articulation, and sometimes there are some difficulties, sometimes things tend to go slightly backwards, but we understand that this is part of the process.

As may be seen, integration is a determining factor in the process, both in the sense of the faculty incorporating the contents of the curriculum and with respect to the resistance of the groups to changes, or even university-community integration. The partnership between the medical school and the teaching-services must be improved.

Another component is the lack of financial resources to implement the process of change and to maintain the curricular strategy. This problem is present in several schools, not only in the internal structure but in the overall structure itself and in the partnership between the medical school and the network that provides healthcare to the community.

**E2: **One of the things I consider a problem is the library. I really need to adapt it adequately.

**E3: **We need to identify other sources of aid, principally to be able to offer courses, hire consultancy services, and to stimulate community interaction since we don't have funding to pay people.

**E17: **...we have several operational difficulties because the funding is in a single account belonging to the government, isn't it, belonging to the university; so, everything has to go out to tender, to a bid.

Every process of curricular reform needs funding to implement its new guidelines and to support the structures that have already been put in place.

Another component mentioned was the limitations represented by the faculty. There is a consensus that overcoming these limitations is fundamental for the development of the student. Even considering the transformation in which the faculty becomes an advisor and partner in the learning process, crucial points may be found that interfere in the development of the projected curricular changes. The new view of teaching is not centered on the professor, but instead, he/she becomes a supervisor of the student participating in the teaching process, in which the student will learn through practice.

**E2: **The only problem that we continue to have is with respect to the group of anatomy professors and we are trying to find alternatives through the use of skills laboratories with model parts (...) to see if we can identify some professors who would also agree to adopt this way of working.

**E9: **A department with a long tradition of specialist or hospital activities (...) convincing them of the idea that this has to change (...) is difficult.

**E11: **It means permanent, continuous training, training so that these new methodologies may be successfully developed and implemented, and a new kind of relationship may be established with the students.

In other words, the reform was initiated prior to having the human resources required to carry it out. This is a contradiction that indicates a need to be progressively adapting to changes while the faculty is not yet prepared to implement them. This profile of the faculty has existed for decades and the training of new professors to work within this new curriculum has not yet been clearly established.

The course coordinators have experienced the challenge of creating new ways of doing things without removing the current model, and the faculties tend to offer resistance to incorporating the curriculum changes. The new relationship had not been inserted into the historical constitution of each professor. Therefore, there is a perception that this process could undergo a crisis and will take a long period of time to be implemented.

**E4: **It was and is a difficult process, particularly because you are obliged to witness the transition of different curricula (...) which forced us to go ahead with this process as each term approached.

**E7: **Now, with respect to the change in the theoretical concept, we believe that it is possible to change and we believe that this may take between 10 and 15 years considering that we have been discussing it since the year 2000 (...) the university has a structure that is...rigid and inefficient (...) there is a problem (...) we have not clearly defined the knowledge required to generate competence in a doctor.

**E8: **A process of change, of transformation, is much more complex than you can imagine or than you would envisage when you design a project.

**E11: **Always running the risk of...returning to hegemony and to the traditional ways because they are predominant out there (...) the majority of our professors, almost all of them, were trained in the traditional method, in a traditional view.

Another component of interest was the community-service interaction. One of the main guidelines of PROMED is the diversification of practical scenarios as a strategy in training a professional acting in primary healthcare, in this case, after their graduation, acting as general practitioners in the health system. The partnership between the university and the health unit is an agreement between the university and the municipal council where the schools are located. The students can begin their activities from the first phases in the health units of the district/communities, with knowledge of the territory, the customs, the services carried out in the health units, and the profile of the population. The students carry out the activities supervised by tutors contracted by the university, and they also participate in the activities of attending the patient during the course, gaining knowledge of the real demands of the assistance provided, and experiencing the everyday life of the health professionals in the community in which they attend the users of the health service. Ways to integrate the teaching process with the network of health services are being sought, including offering professionals at the local health units post-graduate courses in strategic areas or in areas lacking professionals trained by SUS. Although essential for medical training, this involves various problems, which, although reported in the other components, deserve further emphasis: problems with relationships, lack of funding, cultural differences, training, etc.

**E3: **The students find working in the community very strange; they question whether they are learning medicine there.

**E5: **Our curriculum was absolutely focused on hospitals. By forcing the student into the healthcare network we thought the result would be a greater focus on doctors working in primary and secondary healthcare.

**E8: **The difficulties are constant...our experience of the partnership...with the city council...made us aware that this partnership must have advantages for both sides.

**E10: **We have had some difficulties in placing our students in the healthcare system. We have succeeded because of our long tradition.

The category referring to limitations may be summarized as: the clear view of the coordinators is that this is a *process *of change. All change generates a certain discomfort and when it is a question of changing paradigms, and carrying out these changes with insufficient funding and with personnel who are not yet prepared for reform, this leads to new ways that are "incomplete, full of imperfections and challenges, because new things that continue to be burdened with the old ones need to be reinvented yet again". However, there is a clear perception that "it is the concrete questions that mobilize people; that create the strength required for interaction and action". You only learn to do things by doing them, and this process is still in its infancy [12].

### Challenges

On the other hand, in contrast to the items presented as limitations, other options exist that are considered requirements that must be achieved. A component presented as a limitation may also be a potential item for change. In this respect, three components were identified: integration, the process itself, and the future, which are clearly characterized in the following dialogues.

**E1: **The most important challenges are: basic-clinical integration, integration between areas of knowledge and the collaboration of the professors in adhering to the proposals and working together to achieve this integration.

**E3: **The greatest challenge is basic-applied integration.

**E14: **Implementation of new teaching strategies in the area of primary healthcare in basic health units.

The challenges were found to be similar in the various medical schools. Through comprehension of the perspectives of all of the structural and ideological reforms it is possible to raise awareness of the importance of the governmental programs and the promotion of the curricular changes through programs such as PROMED.

**E4: **This change also brings us...an opportunity to see a curriculum as dynamic, something that should be undergoing permanent revision and adaptation to requirements and which should be in accordance with the wishes of the people who have participated in this educational experience.

**E12: **I think this is going to be one of the most difficult things in the future (...) it is in the change of the actors involved that we show the importance of the partnership, isn't it?

**E19: **There is a long way to go yet before I can say (...) that it is completely consolidated (...) with respect to the teaching-service relationship; in our case we have a good relationship, but it is not perfect.

The challenges encountered go through the process of restructuring and also that of making the faculty aware of the process itself.

**E16: **It is a long process and is not yet complete, principally because we are changing people, changing ways of being.

## Conclusion

In the movement for change in the training of health professionals in Brazil, the PROMED program was understood as being strong enough to trigger other processes. Nevertheless, it faces difficulties, one of which is that it has failed to predict the need for monitoring and the permanent application of an evaluation program. This evaluation will allow us to verify whether PROMED has been able to instigate this change or whether it lacks the necessary influence to achieve this.

The principal social actors involved in the process are ultimately the coordinators of the courses in which the reforms are being implemented. Within the 19 institutions, a great diversity of universes was found, which, in some way, functions as a synthesis of the opinion of these social actors. What may be deduced is that: the participants understand that this is a process, and is therefore unfinished; there are no protocols indicating how the changes should be carried out; and it will take a long time for the changes to be implemented. The course coordinators understand the limitations, and they have been making efforts to overcome them. They know that this process will trigger future changes in the overall practice of healthcare. It will be other professionals who will manage the healthcare system. Changes will no longer be limited to alterations in appearances, rearrangements of schedules or the introduction of a new course unit. "The pathways that lead to this are not easy; they involve suffering and confusion together with the pleasure of discovering new visions, new possibilities, new solutions" [13].

## Competing interests

This article forms part of a doctoral dissertation, held at the Graduate Program in Child and Adolescent Health of the Faculty of Medical Sciences (FCM), the State University of Campinas (UNICAMP), Brazil.

## Authors' contributions

PAS collected data, drafted the manuscript, made the analysis of the results. AMBZ participated in the orientation and preparation of the manuscript. MAR carried out the analysis of the results and participated in the writing of manuscript. All of the authors have read and approved the final manuscript.

## Pre-publication history

The pre-publication history for this paper can be accessed here:


